# Exposure to two-dimensional ultrathin Ti_3_C_2_ (MXene) nanosheets during early pregnancy impairs neurodevelopment of offspring in mice

**DOI:** 10.1186/s12951-022-01313-z

**Published:** 2022-03-05

**Authors:** Yixian Wen, Le Hu, Jian Li, Yanqing Geng, Yang Yang, Jing Wang, Xuemei Chen, Liliang Yu, Hongyu Tang, Tingli Han, Yongxiu Yang, Xueqing Liu

**Affiliations:** 1 Joint International Research Laboratory of Reproductive and Development, Department of Reproductive Biology, School of Public Health and Management, Chongqing Medical University, Box 197, No. 1 Yixueyuan Road, Yuzhong District, Chongqing, 400016 People’s Republic of China; 2 Department of Obstetrics and Gynecology, Key Laboratory of Gynecologic Oncology of Gansu Province, The First Hospital of Lanzhou University, No. 1 West Donggang Road, Chengguan District, Lanzhou, Gansu 730000 People’s Republic of China; 3 Department of Obstetrics and Gynecology, Prenatal Diagnosis Center, The First Affiliated Hospital of Chongqing Medical University, Chongqing, People’s Republic of China; 4 College of Basic Medicine, Chongqing Medical University, Chongqing, People’s Republic of China; 5 Department of Obstetrics, The First Affiliated Hospital of Chongqing Medical University, Chongqing, People’s Republic of China; 6 Department of Obstetrics and Gynecology, The Second Affiliated Hospital of Chongqing Medical University, Linjiang Road, Yuzhong District, Chongqing, 400016 People’s Republic of China

**Keywords:** Reproductive fitness, Offspring health, Biosafety, Environmental factors, Ti_3_C_2_ nanosheets, Neurotoxic

## Abstract

**Background:**

Two-dimensional ultrathin Ti_3_C_2_ (MXene) nanosheets have been extensively explored for various biomedical applications. However, safety issues and the effects of Ti_3_C_2_ on human health remain poorly understood.

**Results:**

To explore the influence on foetal or offspring after exposure to Ti_3_C_2_ nanosheets, we established a mouse model exposed to different doses of Ti_3_C_2_ nanosheets during early pregnancy in this study. We found that Ti_3_C_2_ nanosheets had negligible effect on the reproductive ability of maternal mice, including average pregnancy days, number of new-borns, and neonatal weight, etc. Unexpectedly, abnormal neurobehavior and pathological changes in the cerebral hippocampus and cortex in adult offspring were observed following Ti_3_C_2_ nanosheet treatment. In further studies, it was found that Ti_3_C_2_ exposure led to developmental and functional defects in the placenta, including reduced area of labyrinth, disordered secretion of placental hormones, and metabolic function derailment. The long-chain unsaturated fatty acids were significantly higher in the placenta after Ti_3_C_2_ exposure, especially docosahexaenoic acid (DHA) and linoleic acid. The metabolic pathway analysis showed that biosynthesis of unsaturated fatty acids was upregulated while linoleic acid metabolism was downregulated.

**Conclusions:**

These developmental and functional defects, particularly metabolic function derailment in placenta may be the cause for the neuropathology in the offspring. This is the first report about the effects of Ti_3_C_2_ nanosheet exposure on pregnancy and offspring. The data provides a better understanding of Ti_3_C_2_ nanosheets safety. It is suggested that future studies should pay more attention to the long-term effects of nanomaterials exposure, including the health of offspring in adulthood, rather than only focus on short-term effects, such as pregnancy outcomes. Metabolomics could provide clues for finding the prevention targets of the biological negative effect of Ti_3_C_2_ nanosheets.

**Graphical Abstract:**

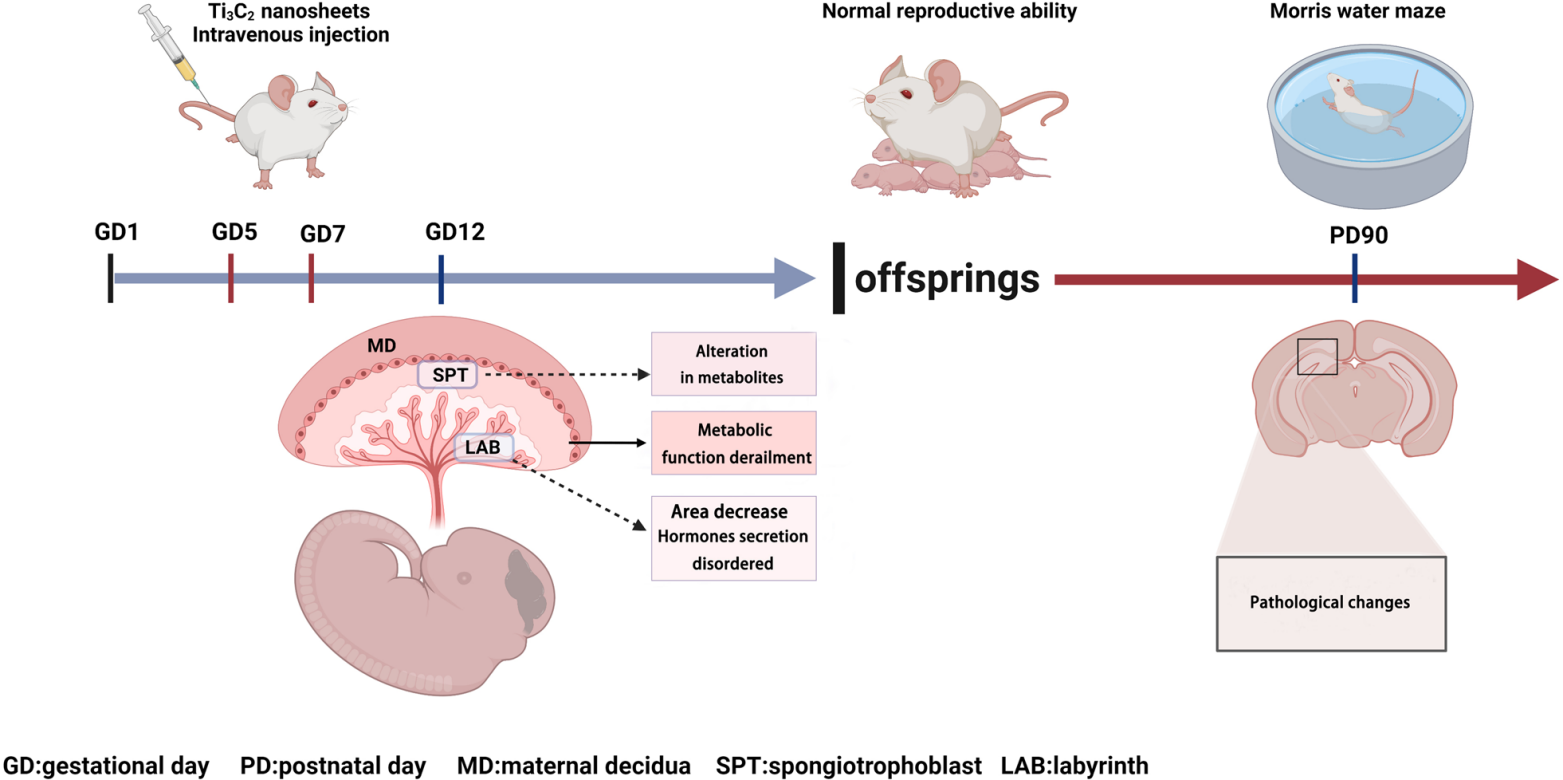

**Supplementary Information:**

The online version contains supplementary material available at 10.1186/s12951-022-01313-z.

## Introduction

Assessment of the detrimental effects of environmental factors on human health and reproductive fitness has attracted much attention in recent years [1]. Most developmental abnormalities in the foetus, especially neurodevelopmental defects in humans, resulting from maternal exposure to toxins during early pregnancy [2]. During early pregnancy, a series of drastic changes occur in the maternal physiology, metabolism, endocrine activity, and immune system, rendering the foetus high sensitivity to environmental factors [3, 4]. The placenta is the key organ between mother and foetus. The proper structure and function the placenta play an important role in foetal organogenesis and neurodevelopment. Impairment of placental structure and function will lead to abnormal pregnancy outcomes such as malformation, miscarriage, and intrauterine growth restriction et al. [5, 6]. With the development of nanotechnology, various nanomaterials have pervaded different aspects of human lives through cosmetics, paints, medical products, textiles, personal care, and other products. The use of these products increases the probability of human exposure to nanomaterials [7]. The increasing application of nanomaterials and their discharge in the environment are posing potentially high risks to the ecological system and human health [8, 9]. More and more evidence shows that exposure of foetuses to nanomaterials can have long-lasting adverse effects on nervous system after birth and into adulthood. For instance, maternal exposure to TiO_2_ nanoparticles (NPs) induced retardation of axonal and dendritic outgrowth in mice offspring [10]. Neurobehavioral defects were found in offspring following pregnancy exposure to carbon black NPs [11].

MXene is a new type of two-dimensional (2D) nanomaterial. Its formula is M_n + 1_X_n_T_x_, where M is a kind of transition metal, X represents carbides, nitrides, or carbonitrides, and T is a surface functionality group. It was derived from the corresponding MAX phase by selective etching of the interlayer Al atoms of a transition metal carbide, Ti_3_AlC_2_ [12]. Owing to unique physicochemical properties, MXene nanosheets have recently gained increased attention in biosensing, photothermal/photodynamic therapy, drug delivery, and wastewater treatment [13]. MXenes were earlier considered non-toxic, biocompatible, and easily biodegradable because they are comprised of Ti, Ta, and Nb, which are inert in biological systems. Besides, nitrogen and carbon are also essential biological elements [14]. Recently, the potential toxicological effects of Ti_3_C_2_ nanosheets on the lung, liver, and immune system have been investigated *in vivo* or *in vitro* [15, 16]. However, experimental research on the biological safety of Ti_3_C_2_ nanosheets are limited. The effects of Ti_3_C_2_ nanosheets during the window of pregnancy exposure are mostly unexplored.

Metabolomics is a powerful approach to detect, identify, and quantify small molecules (below 1 kDa) in biological tissues and has been broadly utilized in toxicological studies for biomarker discovery and potential mechanisms [17, 18]. The metabolism of placenta reflects the physiological and pathological status of the maternal organism as well as changes in response to environmental stimulus, including toxins exposure [19]. As the only interface between the foetal and the maternal circulation, placenta executes multiple functions to maintain normal foetal development, especially brain [20]. Adequate levels of fatty acid in foetal neural membranes are required for maturation of neurovascular coupling and cortical astrocytes. Abnormal placental lipid transport and metabolism causes defects in neural function and behaviour and is associated with learning difficult and emotional alters [21, 22]. Thus, the metabolome of placenta is extremely essential for foetal neurodevelopment.

In the present study, we established a mouse model exposed to different doses of Ti_3_C_2_ nanosheets by tail-vein injection during early pregnancy, and assessed the effects of Ti_3_C_2_ nanosheets on the female reproductive function and long-term effects on offspring. The maternal pregnancy status, the neurobehavioral manifestations of offspring and placenta function that mediated maternal-foetal communication were evaluated. To further elucidate the potential mechanisms, we used metabolomics approaches to identify molecular changes in placenta following maternal Ti_3_C_2_ nanosheets exposure. The results described herein should provide an important reference for the female reproductive toxicity of Ti_3_C_2_ nanosheets, and form an experimental basis for determining the safety of Ti_3_C_2_ nanosheets. This study can also provide meaningful insights into the clinical application of this nanomaterial.

## Materials and methods

### Synthesis and characterization of Ti_3_C_2_ nanosheets

Ti_3_C_2_ nanosheets were obtained by etching Ti_3_AlC_2_, purchased from Jilin 11 Technology Co., Ltd. (Jilin, China), in the HCl/LiF mixture, as reported previously [23]. The HCl/LiF mixture was prepared by mixing 80 mL concentrated HCl and 4.0 g LiF. The mixture was stirred for 30 min until the salt was dissolved. Ti_3_AlC_2_ powder (2.0 g) was added to the mixed solution gradually to avoid violent exothermic reaction and stirred for 24 h at 40 °C. Thereafter, the obtained compound was washed several times with distilled water until the pH of the supernatant was approximately 6. After sonication for 1 h under an Ar flow, the MXene dispersion was collected by centrifugation at 3500 rpm for 1 h. The degradation of Ti_3_C_2_ nanosheets was performed for intravenous injection, as described previously [24]. The ultrastructure and morphology of Ti_3_C_2_ nanosheets were examined by atomic force microscopy (AFM, Multimode 8, Bruker, USA), scanning electron microscopy (SEM, JSM 6701 F, JEOL, Japan), transmission electron microscopy (TEM, JEM-2100FEG, JEOL, Japan), X-ray photoelectron spectroscopy (XPS, ESCALAB 250Xi, Thermo Fisher Scientific, USA), and X-ray diffraction (XRD, X’PERT PRO, PAN-analytical).

#### Animal model

Adult, virgin female, and fertile male mice (KM-strain, 28–30 g) were purchased from Chongqing Medical University Lab Animal Centre (Certificate: SICXK (YU) 2007-0001) and were used by the Institutional Animal Care and Use Committee of Chongqing Medical University (Chongqing, China). All the mice were housed in a consistent environment, with positive-pressure air-conditioned units and maintained at 22 °C± 2 °C (relative humidity, 55% ± 10%) on a 12-h light/dark cycle, and provided forage and water *ad libitum*. Female mice were mated with males at a 2:1 ratio overnight, and the appearance of the vaginal plug was considered as the gestational day (GD) 1.

Based on the research of Ti_3_C_2_ nanosheets as a novel photothermal agent for cancer therapy, we chose half of the reported lowest dose as the highest dose per day in the present study [22]. To find a safe dose range for the pregnant stage, we selected half of the highest dose as the intermediate dose and 10% of the highest dose as the lowest dose. Pregnant mice were divided randomly into the following five groups: (1) negative control group, (2) vehicle control group, (3) 0.5 mg/kg/day Ti_3_C_2_ nanosheets group, (4) 1.25 mg/kg/day Ti_3_C_2_ nanosheets group, (5) 2.5 mg/kg/day Ti_3_C_2_ nanosheets group, with 12–15 mice per group. The volume of Ti_3_C_2_ nanosheet suspension used for exposure was 0.05 mL/10 g body weight. Due to the high sensitivity to environmental factors during early pregnancy, we injected mice intravenously from GD5 when the blastocyst initially implants into the uterus for 3 consecutive days. The maternal body weight was recorded during the placenta development (GD5-12). The serum, uterus, and vital organ tissues were collected from pregnant mice on GD8, GD9, GD10, and GD12. After dissecting, the placenta and foetus were separated from the uterus on GD12 and weighed individually. Within the first 2 days after birth, the days of pregnancy, number of foetuses, and birth weight were recorded. The Morris water maze and elevated plus maze tests were conducted on postnatal day 90. In total, elevated plus maze and Morris water maze tests were conducted in 10 adult offspring from each dose group, the morphological changes were detected then.

### Accumulation of Ti_3_C_2_ nanosheets in vivo

The uterus tissues were collected on GD8, GD9, and GD10, and the placenta and foetus were separated from the uterus on GD12. The detection of Ti_3_C_2_ accumulation *in vivo* was performed by Beijing Zhongkebaice Technology Co., Ltd. Titanium (Ti) content in tissues was measured by Inductively Coupled Plasma Mass Spectrometry (ICP-MS, 7800, Agilent Technologies Inc., USA).

### Analysis of the spatial learning and memory abilities in offspring mice

Morris water maze test was conducted to examine the spatial learning and memory of offspring at the age of 90 days after maternal exposure to Ti_3_C_2_ nanosheets on GD5-7. The Morris water maze consisted of a circular pool (150 cm in diameter and 50 cm in height) filled with tap water up to 30 cm and maintained at 25 °C ± 2 °C. The pool was divided into four quadrants and a circular escape platform (9 cm diameter) was placed 1.5 cm below the surface of the water in the centre of a specific quadrant. A camera was installed on the ceiling to record the swim path of each mouse with the ANY-maze software. After the first acclimation to the maze during a one-trial habituation period, all the mice were first habituated to the maze with a 120 s swim in the pool, and the pool was made opaque by addition of a non-toxic water-soluble black paint. The directional navigation test began the day after habituation. Mice were subjected to five training sessions daily, for 5 consecutive days. For each trial, all the mice were free to search for the platform for 60 s. Once the mice reached the platform, escape latencies were recorded and it was allowed to remain on the platform for 10 s. If a mouse failed to reach the platform within 60 s, it was gently placed on the platform by the experimenter for 10 s. During the trials, the mice were towelled and dried and kept at rest for at least 1 h. The escape latency was calculated as the average of total time taken in all trials per day.

On the sixth day of trial, mice performed a 60 s probe trial in which the platform was removed. Each mouse was released in the water, starting from the quadrant opposite to the platform quadrant, and the swimming time was tracked for 60 s. The number of cross-platform and routes for each mouse was recorded during the 60 s.

### Analysis of the behaviour of offspring

Elevated plus-maze test was performed to assess the behaviour of the offspring. The apparatus consisted of two open arms (50 × 10 × 0.5 cm) and two closed arms (50 × 10 × 40 cm) arranged in a cross-like disposition, and a central platform (10 × 10 cm). The entire apparatus was made of dark polyvinyl plastic and was elevated 55 cm above the floor. Each mouse was placed in the central platform facing the same closed arm and could explore the apparatus freely for 5 min. The time spent on and the number of entries of the four paws into the open and closed arms were recorded. The percent open arms time spent was calculated as the time spent in the open arms per total time. The percent number of entries into the open arms was calculated as the number of entries into the open arms divided by the total number of entries into the four arms.

### Analysis of morphological changes

Fresh tissues, including heart, liver, spleen, lung, kidney, ovary, uterus, placenta, and brain were fixed in 4% paraformaldehyde for 4–6 h at 26 °C The tissues were dehydrated with a gradient series of alcohol and embedded in paraffin (melting point, 62 °C). Sections were cut (5 μm thick) for haematoxylin and eosin (H&E) staining and visualized under an Olympus microscope (BX40, Olympus, Tokyo, Japan). Representative images of general morphology of tissues were captured using an iPhone (iPhone 7 plus, Apple, USA). The images were analysed using the cell Sens Standard software (Olympus, Tokyo, Japan).

### Analysis of the placental development

The analysis was performed by detection of several important proteins using immunohistochemistry. Briefly, the tissue sections were deparaffinized in xylene preheated at 60 °C for 30 min and hydrated with a gradient series of alcohol. After antigen retrieval by boiling in sodium citrate buffer for 15 min, endogenous peroxidase activity was quenched by incubation with 1% H_2_O_2_ for 10 min, and then blocking was performed with 10% goat serum for 15 min at 26 °C. Thereafter, the sections were incubated with primary antibodies, including VEGFR2 (1:800, Cell Signalling Technology, Inc., MA, USA), Ki67 (1:200, Abcam Inc., Cambridge, MA, USA), BMP2 (1:100, Abcam Inc., Cambridge, MA, USA), and CK8 (1:200, Abcam Inc., Cambridge, MA, USA) at 4 °C overnight, and subsequently with secondary antibodies (goat anti-rabbit IgG or goat anti-mouse IgG) at 1:1000 dilution (Santa Cruz Biotechnology Inc., CA, USA) for 30 min at 37 °C. After washing three times with phosphate-buffered saline (PBS), the sections were incubated in 3,3′-diaminobenzidine for 2 min and were finally stained with haematoxylin for 1 min and visualized under the Olympus microscope.

### Analysis of the endocrine function of the placenta

Enzyme-linked immunosorbent assay (ELISA) test kits (BD, USA) were used for determining the levels of β-chorionic gonadotrophin (β-CG), and placental growth factor (PlGF) and its soluble receptor (sFlt-1) in the serum. The assays were performed by Shanghai Yanhui Biotechnology Co., Ltd. using a Microplate Analyzer (Rayto RT-6100, China).

### Analysis of the metabolite profiles

We used two different methods to extract metabolites from placental tissues and serum. 50 mg placental tissues were extracted with 500 µL of cold methanol-water (50% v/v) containing 20 µL of 2,3,3,3-d4-alanine (10 Mm). After homogenized and centrifuged (17,000*g*, 15 min), the supernatant was dried for derivatization. 600 µL cool methanol was added into 150 µL serum, and 20 µl 2,3,3,3-d4-alanine (10 Mm) as internal standard was added. vortex for 1 min, and then incubate at − 20 °C for 30 min. The plasma supernatant was collected for derivatization after centrifugation (17,000*g*, 15 min). The methyl chloroformate (MCF) method were performed based on the protocol published by Smart et al. [25]. Briefly, 200 µL of sodium hydroxide (1 M), 200 µL methanol and 34 µL of pyridine were added to dried sample. Then 20 µL of MCF were added with 30 s of oscillation and another 20 µL of MCF was subsequently added with 30 s of oscillation. Afterward, 400 µL of chloroform and 400 µL of sodium bicarbonate were added and oscillated for 10 s respectively. After centrifuging at 2000 rpm for 10 min at 4 ℃, anhydrous sodium sulphate was added to remove excess water and the lower chloroform phase was isolated for gas chromatography–mass (GC–MS) analysis. The derivatized samples were detected by the GC7890 system with an MSD5975 mass selective detector (Agilent, USA). The GC–MS analysis conditions were performed as in our previous study [26]. Automated Mass Spectral Deconvolution & Identification System V2.1 software (AMDI S, National Institute of Standards and Technology, USA) was used for metabolite deconvolution and identification.

### Statistical analysis

The GraphPad Prism version 8.0 software (Graph-Pad Software Inc., La Jolla, CA, USA) was used for data analysis with Student’s *t*-tests used for data comparison for two groups and two-way analysis of variance (ANOVA; Kruskal-Wallis test) used for comparisons of multiple groups. A value of p < 0.05 was considered significant. All data are reported as mean ± standard deviation (SD). Principal component analysis (PCA) was conducted using the mixOmics R-package [27]. A Student’s t-test and false discovery rate (FDR) were executed in R to compare metabolites between control group and different dose groups. The area under the receiver operating characteristic (ROC) curve was performed using the pROC R-package [28].

## Results

### Characterization of Ti _3_C_2_ nanosheets

**Fig. 1:**
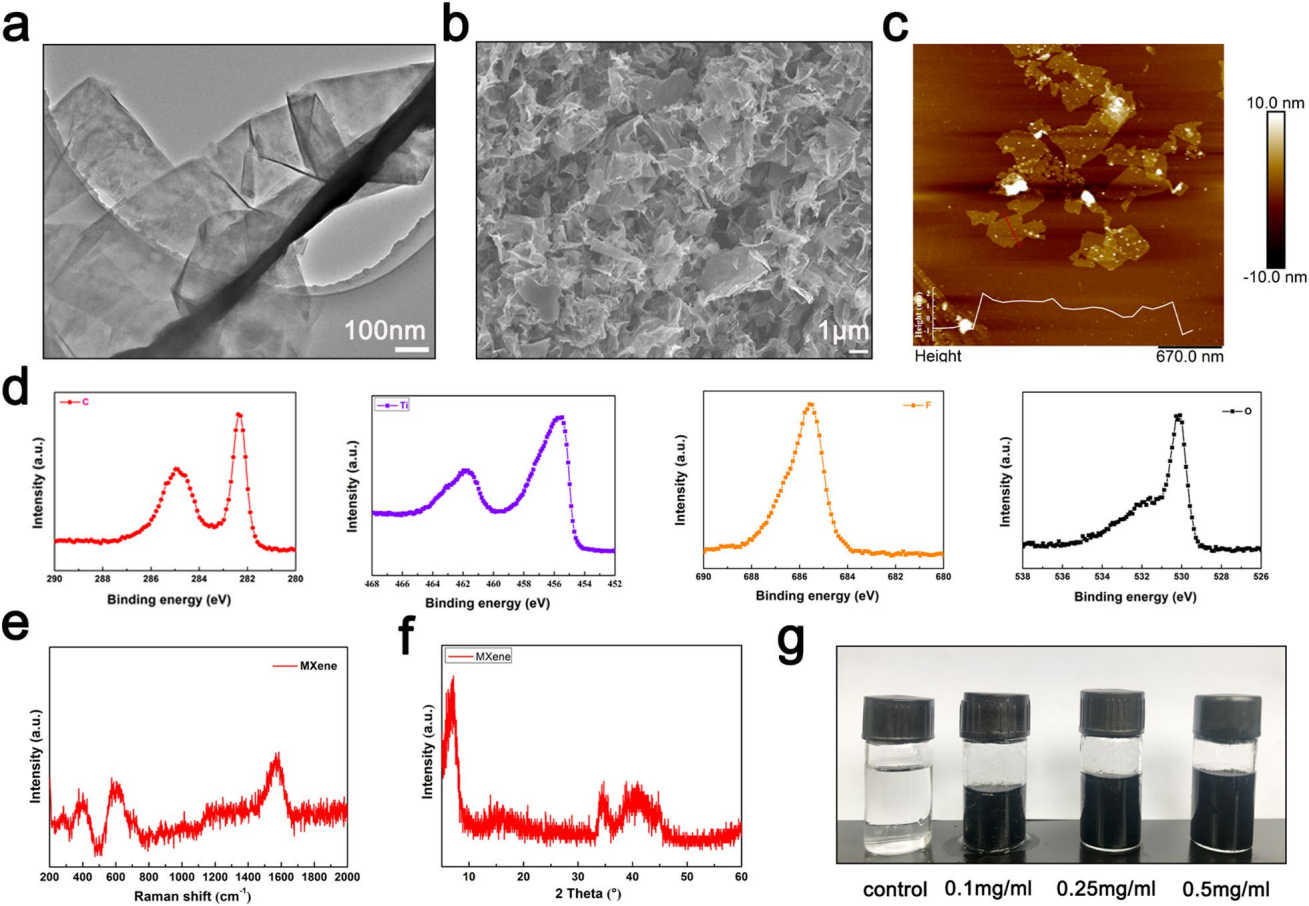
Characterization of Ti_3_C_2_ nanosheets. **a** TEM image of single-layer Ti_3_C_2_ nanosheets. **b** SEM image of Ti_3_C_2_ nanosheets. **c** AFM image of Ti_3_C_2_ nanosheets. **d** XPS spectrum of Ti_3_C_2_ nanosheets in the C, Ti, F, and O core-level regions. **e** Raman spectrum of Ti_3_C_2_ nanosheets. **f** XRD pattern of Ti_3_C_2_ nanosheets. **g** Dispersibility and stability of Ti_3_C_2_ nanosheets at different concentrations

The morphology of Ti_3_C_2_ nanosheets was assessed by TEM and SEM; the lateral size of the ultrathin nanosheet structure was about 1–2 μm (Fig. 1a, b). The thickness of well-dispersed nanosheets determined by AFM analysis was about 2–3 nm (Fig. 1c). Furthermore, the chemical state and elemental composition were analysed by XPS (Fig. 1d), which revealed characteristic peaks in the C, Ti, F, and O core-level regions. The Raman spectra of Ti_3_C_2_ nanosheets exhibited three peaks at around 400, 600, and 1580 cm^−1^, composited to the G band of the carbon structure (Fig. 1e). In the XRD pattern, the (002) peak of Ti_3_C_2_ nanosheets implied that the Ti_3_AlC_2_ phase was completely converted into Ti_3_C_2_ nanosheets (Fig. 1f). Various concentrations of Ti_3_C_2_ nanosheet solution showed good dispersibility and stability (Fig. 1g).

### Distribution of Ti_3_C_2_ nanosheets in intrauterine tissues

**Fig. 2:**
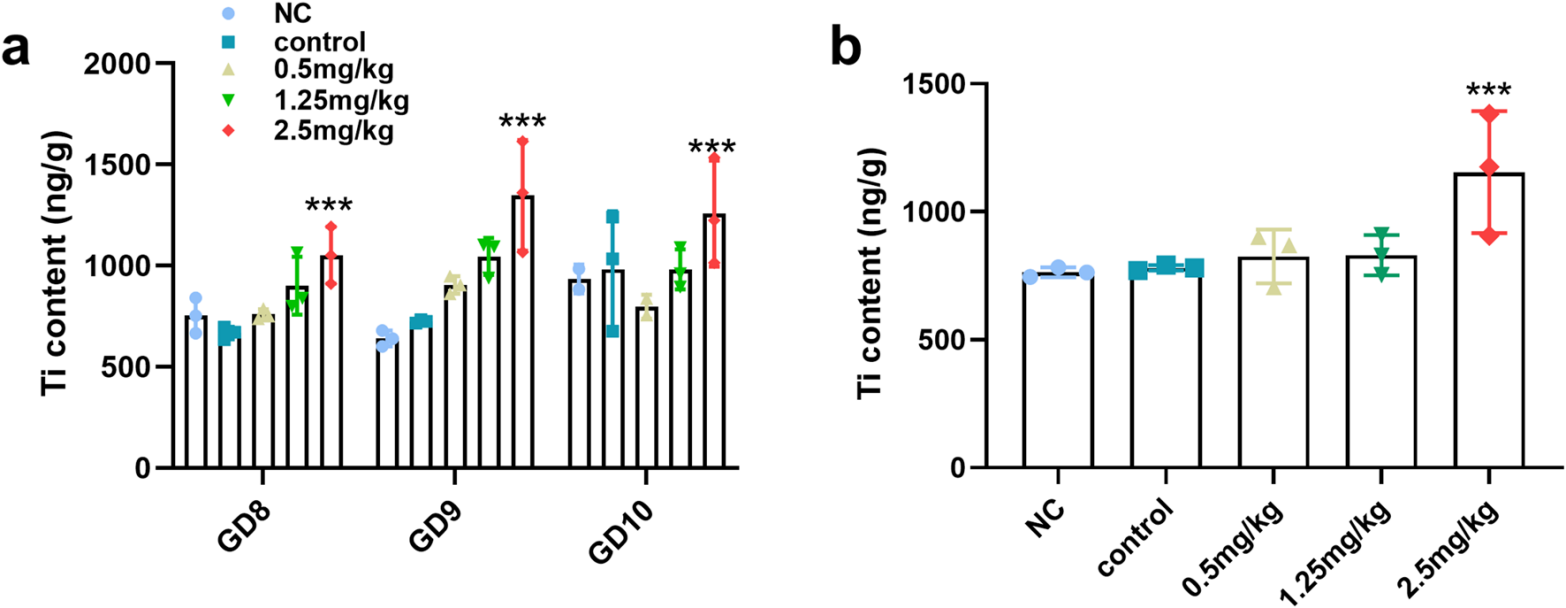
Accumulation of Ti in maternal organs after intravenous injection of Ti_3_C_2_ nanosheets into pregnant mice for 3 consecutive days. **a** The accumulation of Ti in the uterus on GD8, GD9, and GD10. **b** The accumulation of Ti in the placenta on GD12. 0.1–0.15 g tissue in each dose group was used for ICP-MS analysis on different pregnant days. The data were analysed by two-way ANOVA. Data in a and b are shown as means ± SD (n = 3 mice per group). ***p < 0.001 vs. NC group

ICP-MS analysis was performed to detect the biodistribution of Ti in the uterus and placenta. As shown in Fig. 2a, Ti was rapidly accumulated in the uterus after injection of different doses into pregnant mice. The Ti content was increased in the uterus in the 2.5 mg/kg group. Furthermore, Ti was significantly augmented (p < 0.05) in the placenta compared with that in the negative and vehicle control groups after intravenous injection of Ti_3_C_2_ nanosheets in the 2.5 mg/kg group (Fig. 2b). These findings confirm that after intravenous injection of Ti_3_C_2_ nanosheets into pregnant mice, the nanosheets accumulate in the uterus and placenta.

### Exposure to Ti_3_C_2_ nanosheets did not affect the reproductive ability of mice

**Fig. 3:**
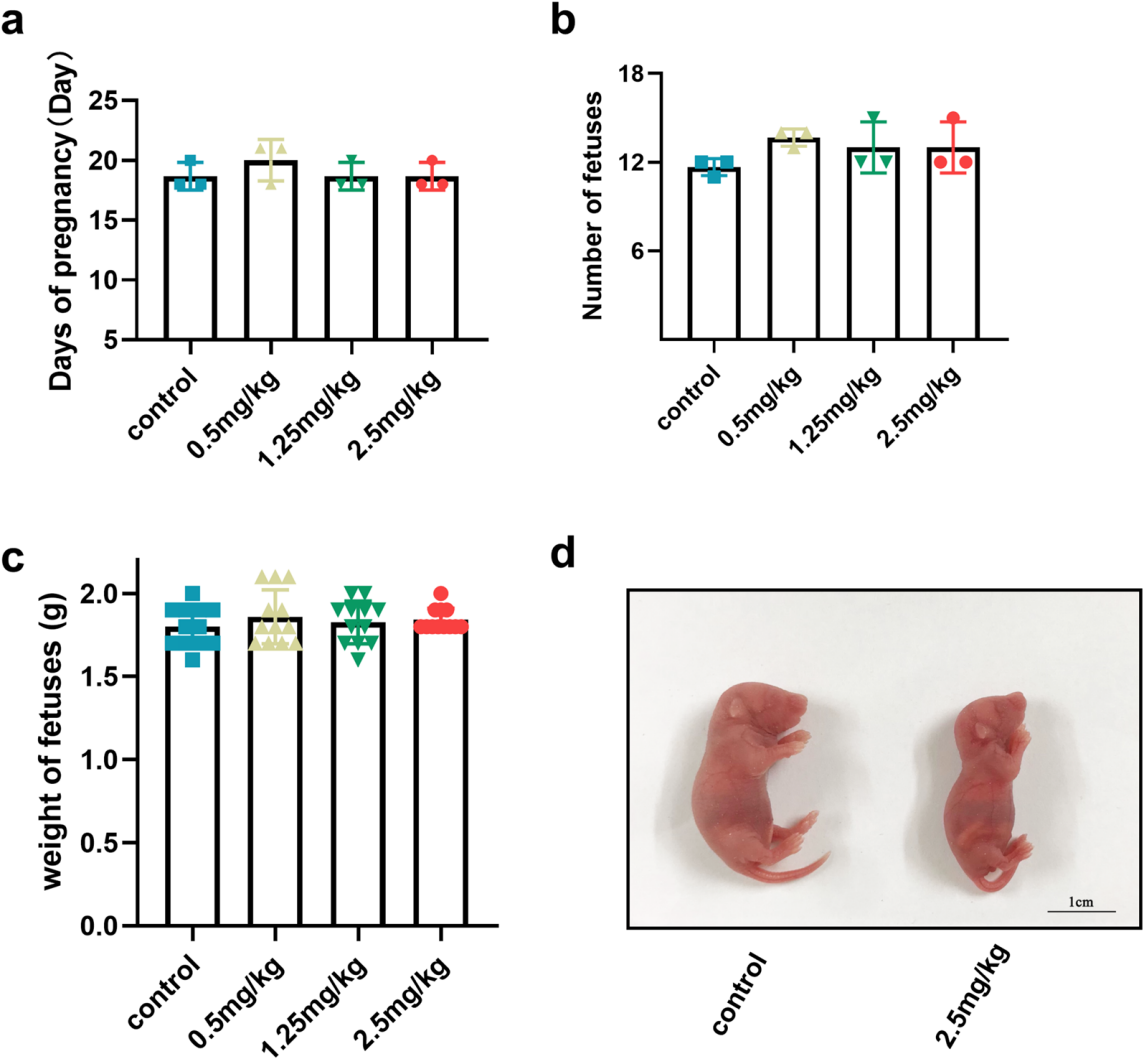
The reproductive ability after intravenous injection of Ti_3_C_2_ nanosheets into pregnant mice for 3 consecutive days. **a** The average pregnancy days at various doses (n = 3 mice per group). **b** The number of foetuses produced per pregnant mice at various doses (n = 3 mice per group). **c** The average weight of neonates (n = 12 neonates per group). **d** Representative images (Scale bar = 1 cm) of neonates in the 2.5 mg/kg group and vehicle control group. Data in **a–c** are shown as means ± SD

To clarify the effect of Ti_3_C_2_ nanosheet exposure on reproduction, we first determined the pregnancy outcome of mice. The average pregnancy days, the number of foetuses produced by each female mouse, and the average neonatal weight were similar to those in the vehicle control group (Fig. 3a–c). Moreover, there was no significant malformation of the foetus at a dose of 2.5 mg/kg (Fig. 3d). These data suggested that exposure to Ti_3_C_2_ nanosheets during early pregnancy did not cause reproductive disability in pregnant mice.

### Exposure to Ti_3_C_2_ nanosheets induced neurobehavioral abnormalities in offspring

**Fig. 4:**
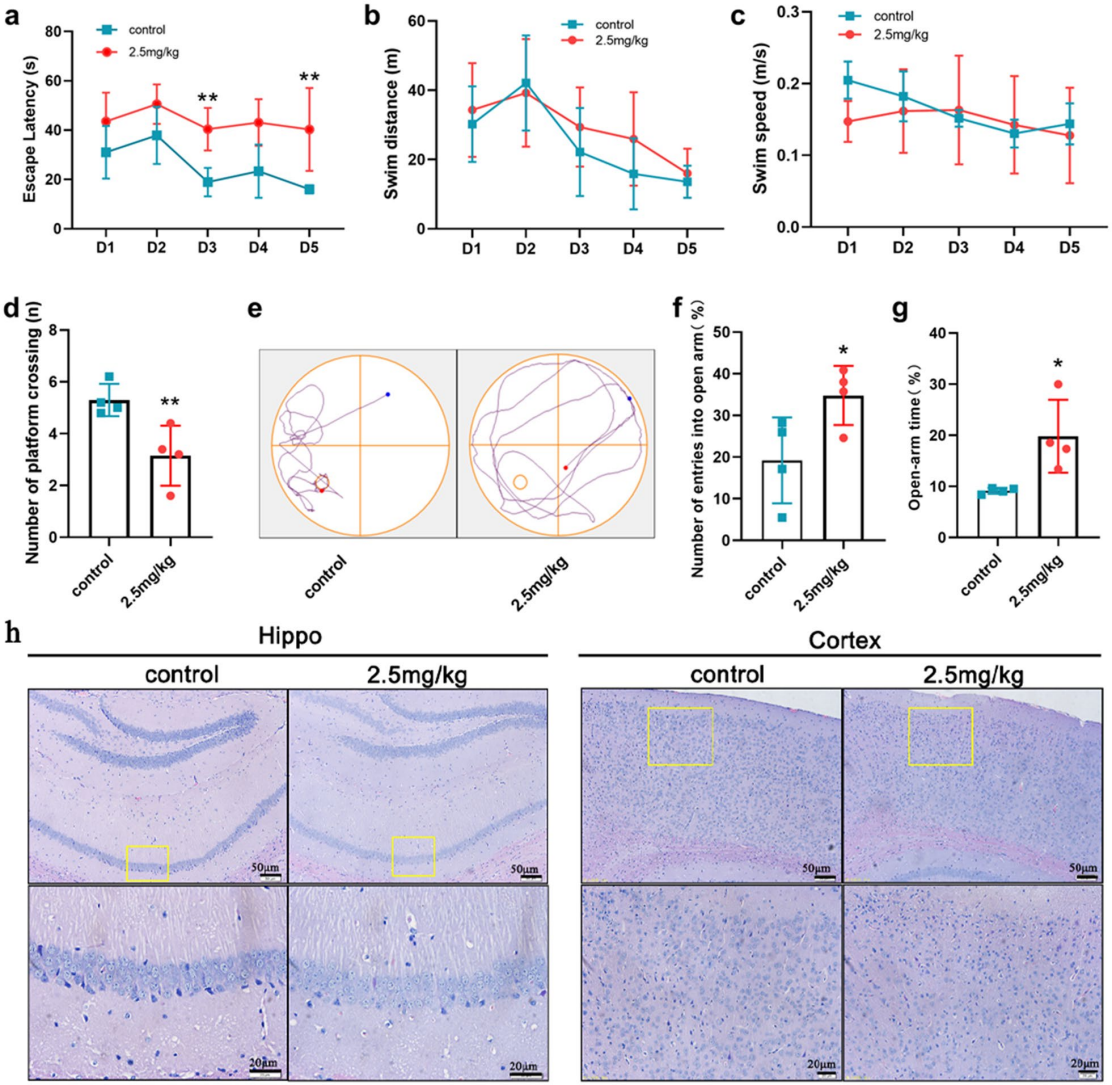
Changes in the neurobehavior and brain morphology of offspring after maternal exposure to Ti_3_C_2_ nanosheets. **a** The escape latency to the hidden platform during the directional navigation test period. **b** The total swim distance per day during the directional navigation test. **c** The average swim speed per day during the directional navigation test. **d** The number of platform crossings during the spatial probe trial. **e** Representative images of offspring in the spatial probe trial. **f** The percentage of the number of entries into the open arm. **g** The percentage of the time stays in the open arm. **h** Representative photomicrographs of morphological changes in the hippocampus and cortex (scale bars = 50 μm and 20 μm). Each experiment was repeated at least three times. Data in **a–g** are shown as means ± SD (n = 4 mice per group). *p < 0.05, **p < 0.01 vs. Control group

To evaluate the neurodevelopment in offspring after maternal exposure to Ti_3_C_2_ nanosheets, Morris water maze and elevated plus maze tests were used as behavioural tasks to evaluate the spatial learning, memory, and behavioural abilities of the offspring mice. As shown in Fig. 4a, the average escape latency in the 2.5 mg/kg group was significantly increased compared with that in the control group during 5 days of directional navigation test. There was no significant difference in the total swim distance and average swim speed between the two groups (Fig. 4b, c). Moreover, the number of platform crossings in the 2.5 mg/kg group was significantly decreased compared with that in the control group in the spatial probe trial (Fig. 4d, e). In the elevated plus-maze, mice in the 2.5 mg/kg group displayed a significant increase in the percentage of entries into the open arm and in the time spent in open arm compared with those in the control group (Fig. 4f, g). These findings indicated that maternal exposure to Ti_3_C_2_ nanosheets impaired cognitive function and had enduring effects on the emotional behaviour of offspring.

To further determine the neurodevelopment of offspring, the morphological changes in the brain tissues were measured by H&E staining. Most neurons in the hippocampal region of mice in the control group were normal, aligned properly with a large and round nucleus, and had a clear nucleolus (Fig. 4h). In contrast, the density of neurons was significantly decreased in the 2.5 mg/kg group and the neurons were arranged loosely. Simultaneously, morphological changes in the cortex, including neuronal cell loss, nuclei shrinkage, and dark staining of neurons, were observed in the 2.5 mg/kg group, which suggested that maternal exposure to Ti_3_C_2_ nanosheets might have caused neurodegenerative changes in the cerebral hippocampus and cortex of offspring mice.

### Effects of Ti_3_C_2_ nanosheets exposure on pregnancy status

**Fig. 5:**
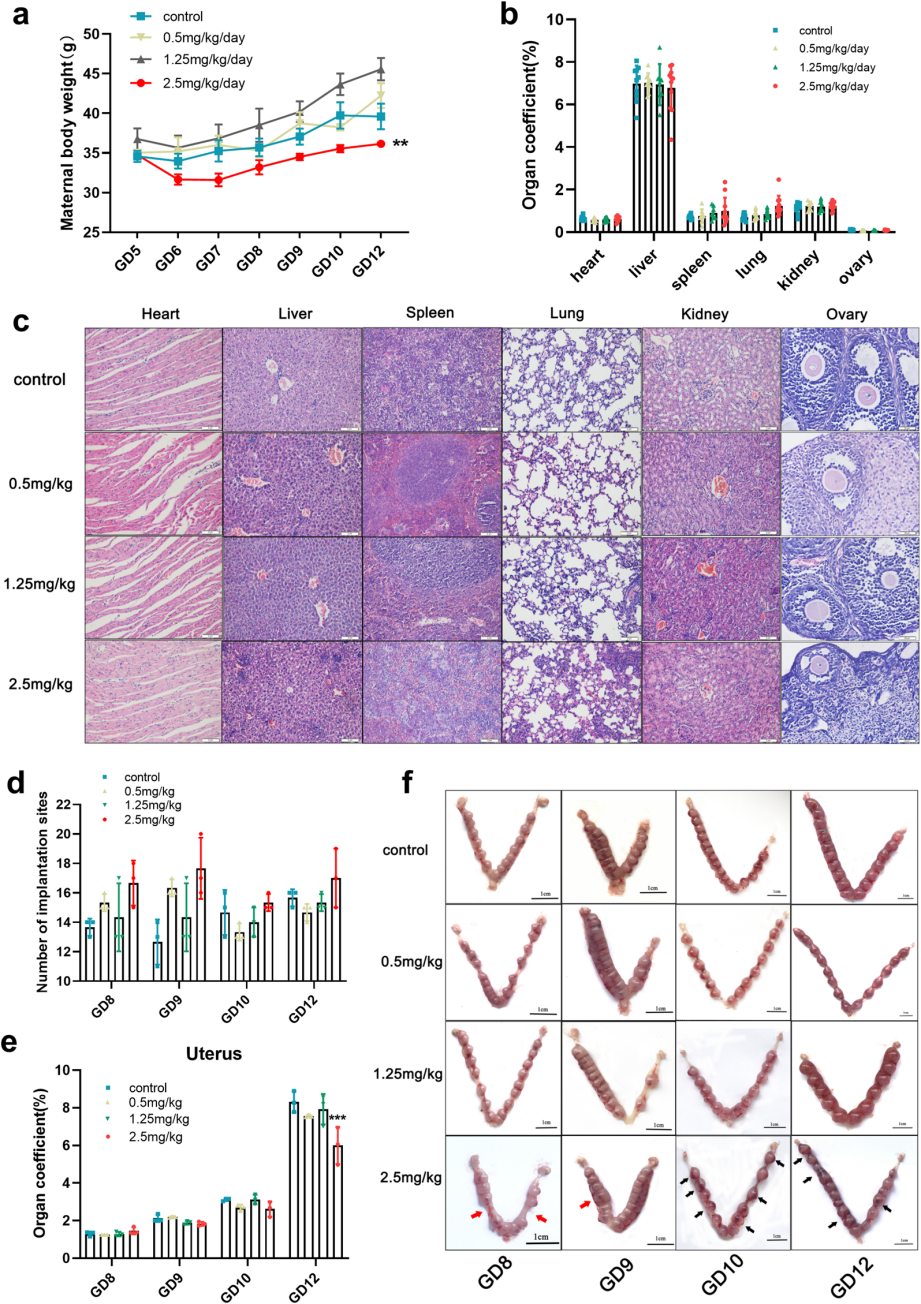
Effects of Ti_3_C_2_ nanosheets on pregnancy status after intravenous injection of various doses. **a** Bodyweight of maternal mice during pregnancy after intravenous injection of Ti_3_C_2_ nanosheets at different doses. **b** The organ coefficient of vital organs on GD12 at different doses (n = 10 per group). **c** Haematoxylin and eosin (H&E)-stained sections (scale bar = 200 μm) showing the histology of vital organs. **d** The number of implantation sites in GD8, GD9, GD10, and GD12 at different doses. **e** The organ coefficient of the uterus on GD8, GD9, GD10, and GD12 at different doses. **f** Representative images (scale bar = 1 cm) of the uterus were collected on GD8, GD9, GD10, and GD12. The data were analysed by one-way ANOVA. Data in b–e are shown as means ± SD (n = 3 mice per group). **p < 0.01, ***p < 0.01 vs. Control group

To trace the causes of neurodevelopmental abnormalities in the offspring after maternal Ti_3_C_2_ nanosheet exposure, we examined the pregnancy status on consecutive days after injection. The maternal body weight was recorded from GD5 to GD12. The maternal body weight showed a slow gain at 2.5 mg/kg dose compared with the gain in other dose groups (Fig. 5a). Next, we calculated the vital organ coefficient (heart, liver, spleen, lung, kidney, and ovary), and observed the micromorphology of vital organs by H&E staining (Fig. 5b, c). No abnormalities were seen at the selected doses. The number of implantation sites had no significant difference on GD8, GD9, GD10, and GD12 at different doses (Fig. 5d). However, compared with the organ coefficient of the uterus in the vehicle control group, an obvious decrease was observed in the 2.5 mg/kg group on GD12 (Fig. 5e). Uneven embryo spacing (red arrows) and uterine bleeding (black arrows) were the observed in 2.5 mg/kg group, especially on GD12 (Fig. 5f). Such changes suggest that the intrauterine environment during pregnancy is more susceptible to interference from external factors.

### Exposure to Ti_3_C_2_ nanosheets impaired the development and function of the placenta in mice

**Fig. 6:**
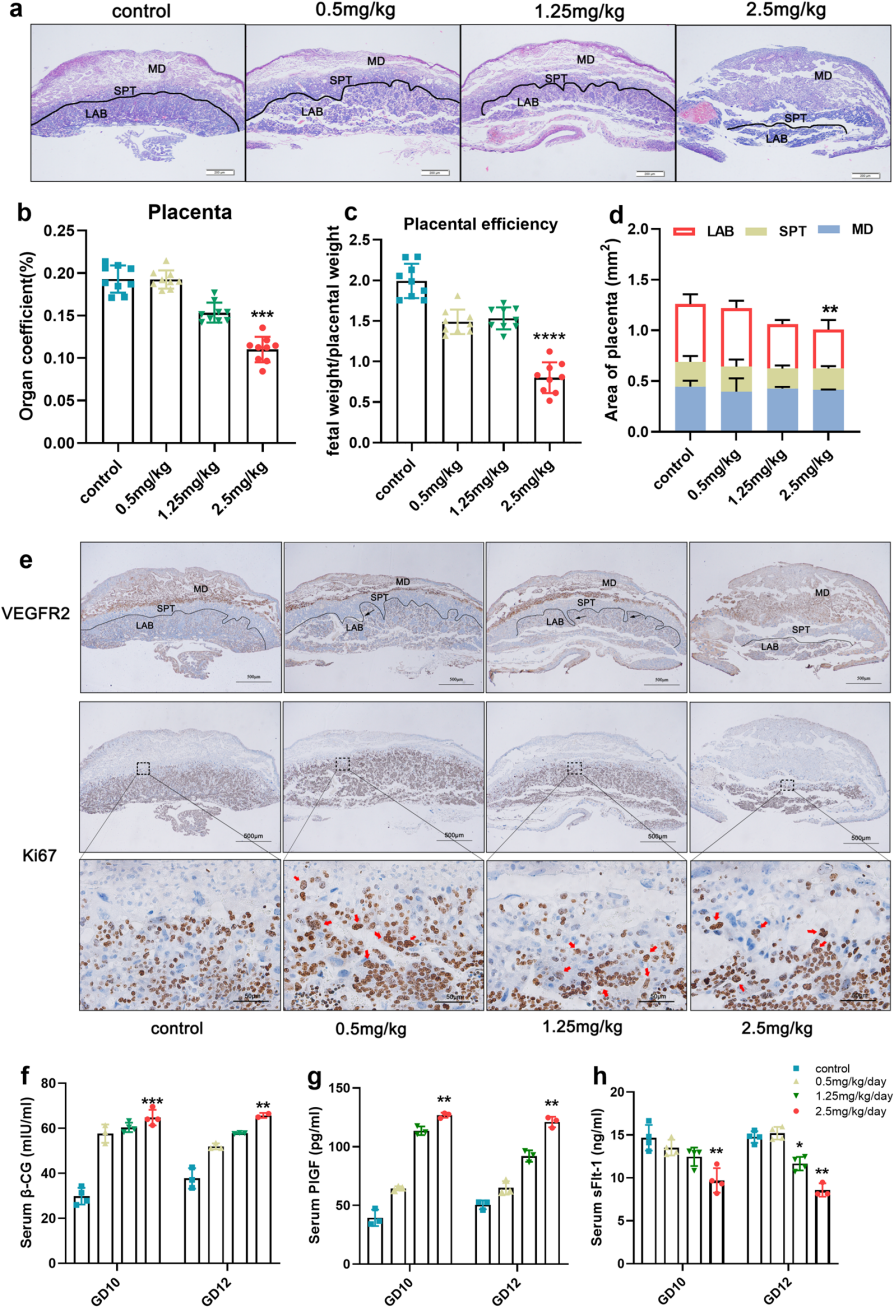
Effects of Ti_3_C_2_ nanosheets on trophoblast proliferation and function of syncytiotrophoblasts. **a** Histological sections and H&E staining (scale bar = 200 μm) of placenta tissue collected on pregnant day 12. LAB, labyrinth; SPT, spongiotrophoblast; MD, maternal decidua. **b** The organ coefficient of placenta on pregnant day 12 at different doses (n = 9 per group). **c** The placental efficiency on GD12 at different doses (n = 9 per group). **d** Quantification of the LAB area, SPT area, and MD area of three placentae per dose. **e** Histological sections and immunohistochemistry staining for VEGFR2 and Ki67 (scale bars = 500 μm and 50 μm) on GD12 at each dose. **f** The levels of serum β-CG on GD10 and GD12 at different doses (n = 3 mice per group). **g** The levels of serum PIGF on GD10 and GD12 at different doses (n = 3 mice per group). **h** The levels of serum sFlt-1 on GD10 and GD12 at different doses (n = 3 mice per group). Data were analysed by two-way ANOVA. Data in **b–h** are shown as means ± SD. **p < 0.01, ***p < 0.001, ****p < 0.000 vs. Control group

The placenta is the key organ that mediates the communication between mother and foetus, therefore, we hypothesized that exposure to Ti_3_C_2_ nanosheets would impair the development of placenta, which might be the main factor in the abnormal neurobehavior of offspring. In the present study, histopathological changes in placenta tissues showed loose structure and haemorrhagic spots in the 2.5 mg/kg group (Fig. 6a). Compared with the vehicle control group, the organ coefficient of the placenta, placental efficiency, and the entire labyrinth surface area quantified on histological sections (n = 3) were significantly decreased (Fig. 6b–d).

To investigate the labyrinth structure in detail, we performed immunohistochemistry for placental artery endothelial cell-specific protein, VEGFR2 (also called kinase insert domain receptor/KDR) [29]. The results showed wedges of spongiotrophoblast invading into the labyrinth in placentae treated with Ti_3_C_2_ nanosheets on GD12 (Fig. 6e, black arrows). Immunohistochemistry for Ki67 showed that more proliferating spongiotrophoblasts were presented in placentae exposed to Ti_3_C_2_ nanosheets than those of control (Fig. 6e, red arrows). To explore the impaired development of placental labyrinth structure and its dysfunction, we performed immunohistochemistry for the bone morphogenetic protein-2 (BMP2), which is required for trophoblast differentiation and spontaneous formation from embryoid bodies [30]. As shown in Additional file 1: Fig. S1a, after exposure to Ti_3_C_2_ nanosheets, BMP2 did not express in the ectoplacental cone (EPC) on GD8. Next, we examined the expression of CK8 (immunohistochemistry staining of trophoblast cells in chorion at pregnant day 9). The result showed that after exposure to Ti_3_C_2_ nanosheets, the thickness of chorion was less than that in the vehicle control group (Additional file 1: Fig. S1b).

Simultaneously, we found that compared with that vehicle control group, the level of serum β-CG was significantly increased in Ti_3_C_2_ nanosheets exposure groups on GD10 and GD12 (Fig. 6f). The trophoblast also secretes placenta growth factor (PlGF) and its soluble receptor (soluble fms-like tyrosine kinase 1, sFlt-1). We found that the level of PIGF was dose-dependently increased, whereas that of sFlt-1 was dose-dependently decreased in groups exposed to Ti_3_C_2_ nanosheets on GD10 and GD12 (Fig. 6g, h).

### Exposure to Ti_3_C_2_ nanosheets changed placental metabolite profiles

**Fig. 7:**
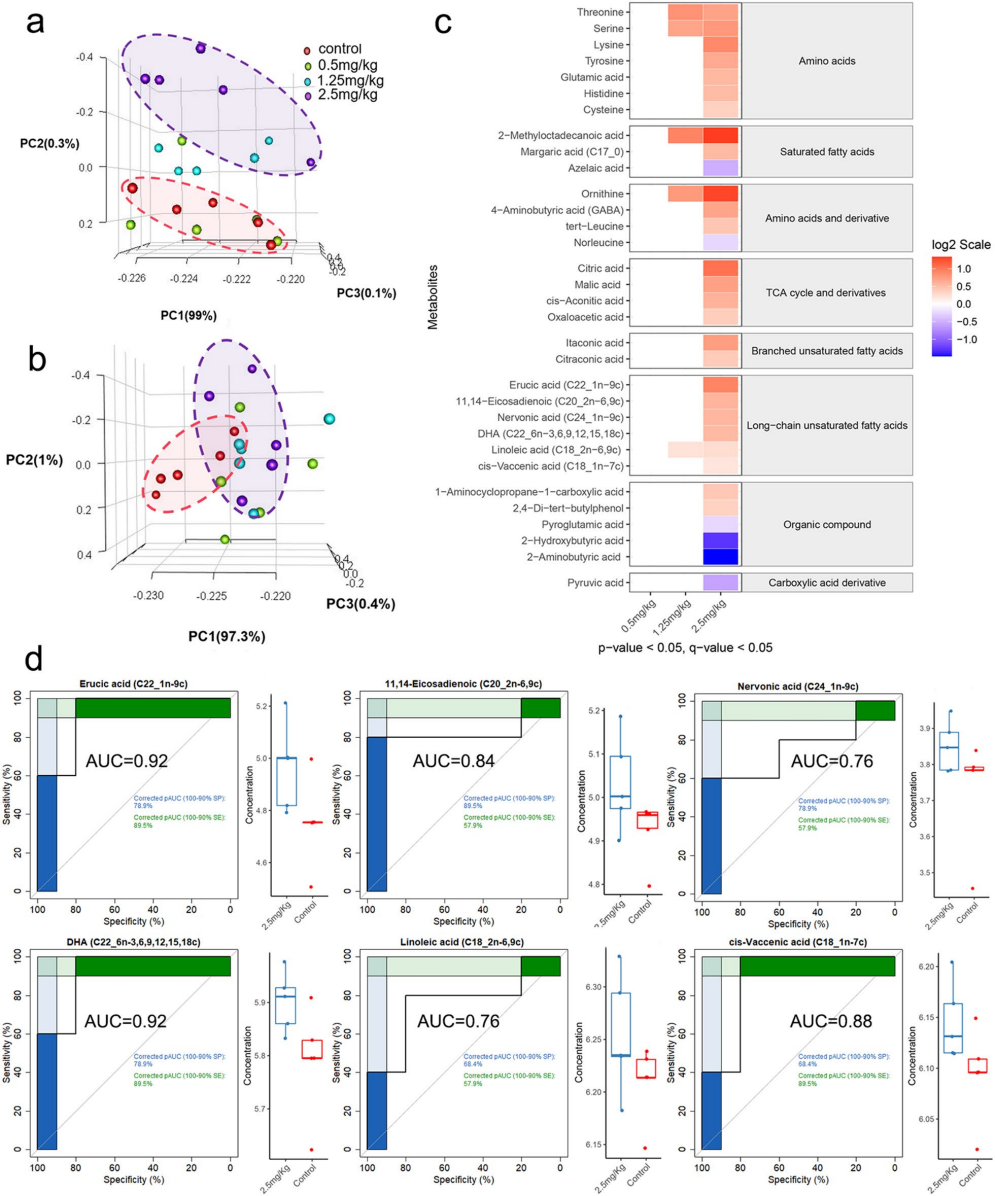
The metabolite profiles of placenta and serum on GD12. **a** Principal component analysis 3D scores plot of placenta at different doses. All samples were clustered into four groups (n = 5 per group). **b** Principal component analysis 3D scores plot of serum on GD12 with different doses. **c** Heat map of the metabolites detected in placenta tissue on GD12. Only the significant metabolites with p < 0.05 and q < 0.05 are displayed in the heatmap. **d** ROC curves for six long-chain unsaturated fatty acids that were detected to have significantly different levels from placental tissue on GD12. *AUC* the area under the curve

To unveil the mechanism of Ti_3_C_2_ nanosheets-induced placenta dysfunction in detail, we analysed the metabolome of placental tissues and the serum on GD12, using gas chromatography–mass spectrometry (GC–MS). A total of 145 metabolites were identified in the placenta, and 152 metabolites were identified in serum. The principal component analysis (PCA) suggested that exposure to Ti_3_C_2_ nanosheets changed metabolites of the placenta in the 2.5 mg/kg group (Fig. 7a). Meanwhile, the metabolites of the serum in the 2.5 mg/kg group didn’t show a distinct disparity from the vehicle control group (Fig. 7b). Compared with the vehicle control group, no metabolites were found to be significantly different in the 0.5 mg/kg group. Only 5 metabolites (Threonine, Serine, 2-Methyloctadecanoic acid, Linoleic acid, and Ornithine) were found to be significantly different in the 1.25 mg/kg group. 32 metabolites were found to be significantly different between the 2.5 mg/kg group and the vehicle control group. These metabolites were composed of a range of amino acids, amino acids and derivatives, saturated fatty acids, branched unsaturated fatty acids, long-chain unsaturated fatty acids, TCA cycle and derivatives, organic compound, and carboxylic acid derivative (p < 0.05 and q < 0.05, Fig. 7c). We also found that the concentration of six long-chain unsaturated fatty acids (11,14-eicosadienoic, cis-vaccenic acid, docosahexaenoic acid, erucic acid, linoleic acid, nervonic acid) was significantly higher in the 2.5 mg/kg group. We performed the receiver operating characteristic (ROC) curve of these six long-chain unsaturated fatty acids which had an area under the ROC curve between 0.76 and 0.92. Both erucic acid and docosahexaenoic acid (DHA) had an area under the ROC curve were 0.92, showed a high level of specificity and sensitivity (Fig. 7d).

### Prediction of the altered metabolic pathway activities in the placenta after exposure to Ti_3_C_2_ nanosheets

**Fig. 8:**
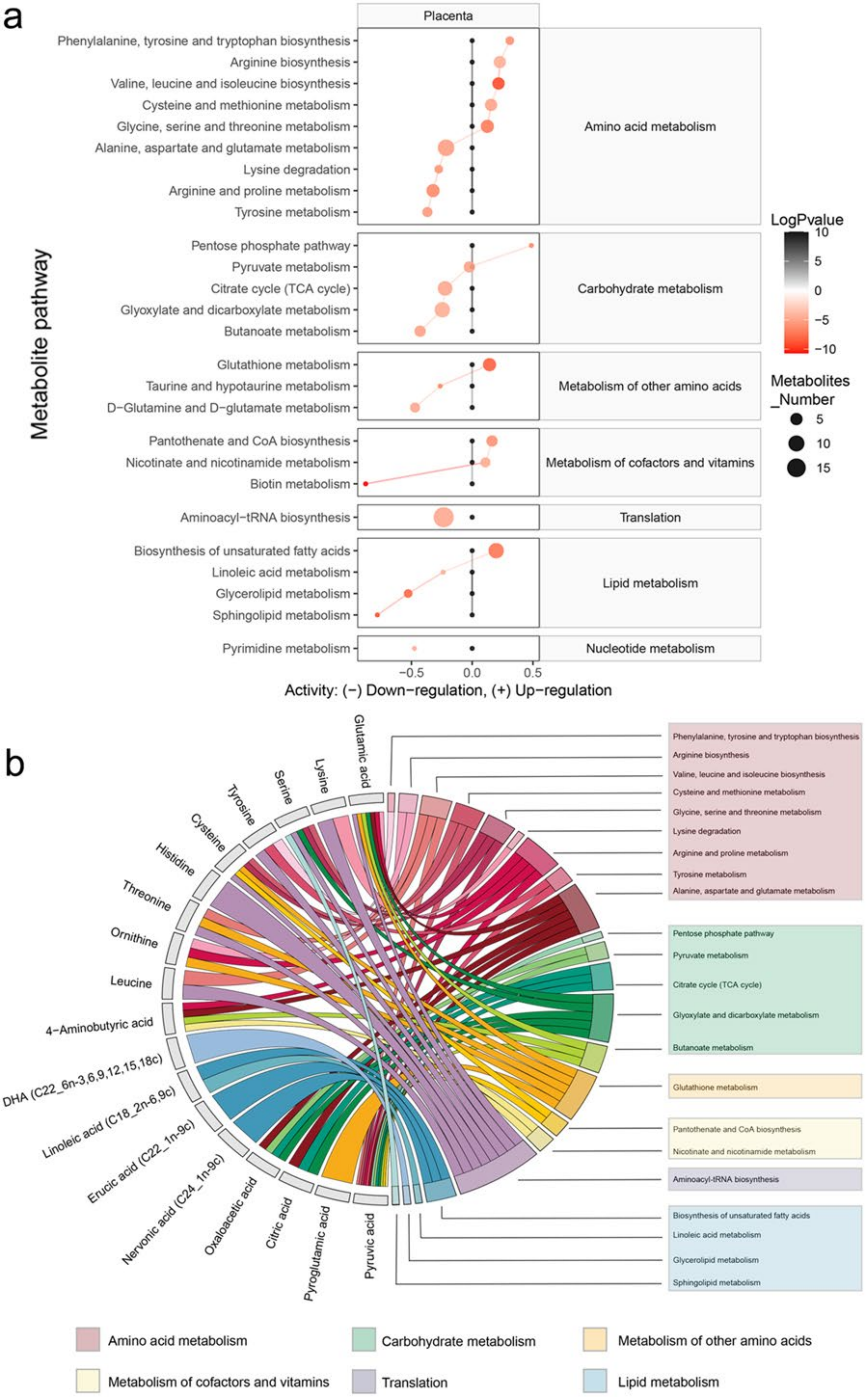
The identified metabolites and the correlated predicted metabolic pathways activities. **a** Predicted metabolic pathway activity for each group. Only the significant metabolites pathway with p < 0.05 and q < 0.05 are displayed in the heatmap. **b** A circle plot displays the detected metabolites that participate in significantly different metabolic pathways

The identified placental metabolites were used to predict the alternation in metabolic pathway activity between the 2.5 mg/kg group and vehicle control group. The KEGG alignment revealed 182 pathways that were linked to the detected metabolites. By contrast, 26 metabolic pathways were significantly altered in the 2.5 mg/kg group. Six amino acid metabolism pathways (phenylalanine, tyrosine and tryptophan biosynthesis, arginine biosynthesis, valine, leucine and isoleucine biosynthesis, cysteine and methionine metabolism, and glycine, serine, and threonine metabolism) were upregulated while four amino acid metabolism pathways (alanine, aspartate, and glutamate metabolism, lysine degradation, arginine and proline metabolism, tyrosine metabolism) were downregulated. In particular, the carbohydrate metabolism (citrate cycle, glyoxylate and dicarboxylate metabolism, and butanoate metabolism) and lipid metabolism (linoleic acid metabolism, glycerolipid metabolism, and sphingolipid metabolism) were significantly downregulated in the 2.5 mg/kg group (Fig. 8a). The correlation between the identified metabolites and the predicted metabolic pathways was illustrated in Fig. 8b. Notably, the lipid metabolism pathways which were participated by four long-chain unsaturated fatty acids including DHA were downregulated.

## Discussion

In recent years, Ti_3_C_2_ nanosheets are gaining wider scientific attention in diverse applications. With the translation of lab experiments into commercial biomedical applications, the biocompatibility and toxicity of Ti_3_C_2_ nanosheets must be stringently assessed. However, the evidence of toxicity caused by Ti_3_C_2_ nanosheets in mammals is still limited. Our study confirmed that after intravenous injection of Ti_3_C_2_ nanosheets into pregnant mice, the nanosheets accumulated in the uterus and placenta. According to previous studies, the nano-size foreign particles can easily cross the blood-placental-barrier (BPB) at the early pregnancy stage because of the lower expression of tight junction proteins in less-developed BPB. For example, CeO_2_ NPs (3–5 nm) accumulated in the placenta after intravenous injection for 3 consecutive days [31], and ZrO_2_ NPs (16 ± 4 nm) crossed the BPB after oral exposure at high doses and then accumulated in the foetal brain [32].

Exposure of the uterus to adverse environmental factors during pregnancy may have profound effects on the health of offspring. According to Barker’s hypothesis, the adaptation of the foetus to the intrauterine environment was considered as the cause of the changes in metabolism, body structure, and physiology [33, 34]. Despite there is no obvious disability on the delivery outcome after exposure to Ti_3_C_2_ nanosheets during early pregnancy, it did not mean the administration of Ti_3_C_2_ nanosheets had no apparent toxicity to pregnant. Ti_3_C_2_ nanosheets accumulated in the placenta after exposure in pregnant mice, which was not only crucial for the pregnancy outcome but also the development of the foetus. Maternal exposure to nanomaterials in early pregnancy is considerably more dangerous for foetal development [35–37]. For instance, maternal exposure to ZrO_2_ NPs affected the tight junction channels, and these NPs crossed the mature BPB and foetal blood-brain barrier (BBB) during the early pregnancy stage [32, 38]. Pregnancy exposure of TiO_2_NPs was reported to deposit in the brain of offspring, causing retardation of axonal and dendritic outgrowth in mouse offspring [10]. Likewise, neurobehavioral impairments emerge in adulthood of offspring resulting from pregnancy exposure to TiO_2_NPs [39]. Maternal exposure to alumina nanoparticles induces abnormal neurobehavior of offspring in the adolescent stage [40], and prenatal exposure to TiO_2_ nanoparticles caused behavioural deficits in offspring [41]. As we all know, spatial learning and memory, and emotional behaviour is the highest level of the integrative function of the brain. The impairment in these complex neurobehaviors was particularly pronounced in adults and became more and more severe over time. Our study demonstrated that there is a long-lasting adverse effect on foetal brain development after maternal exposure to Ti_3_C_2_ nanosheets at an early stage of pregnancy. In this phase, neurulation as a fundamental event of embryogenesis takes place, and eventually, a neural tube which is the predecessor to the brain and spinal cord was formed [42]. As an external and internal harmful factor, Ti_3_C_2_ nanosheets most likely affected this critical process. Accordingly, Ti_3_C_2_ nanosheets shouldn’t be regarded as safe for pregnancy.

As a bridge that exchanges oxygen and nutrients, the placenta is an important organ that supports the survival and growth of the mammalian embryo in the uterine environment, while providing a protective barrier for the foetus [20]. Several researches confirmed that placenta response to environmental stimulus will influence development of foetal brain and postnatal behaviours of offspring [43, 44]. In mice, a fully formed placenta is composed of three key layers: the maternal decidua, the spongiotrophoblast, and the labyrinth [45]. The placental labyrinth initiates to generate on GD7 and then the posterior end of the embryo to extend the extraembryonic mesoderm (allantois) on GD8. Around GD9, the chorion arising from the mesoderm at the posterior end of the embryo contacts with the allantois, termed chorioallantois fusion. After hours of this event, the labyrinth is created by the trophoblast with its associated foetal blood vessels undergoing extensive villous branching on GD12 [20]. In our study, the trophoblast differentiation deficiency on GD8 and thickness of chorion on GD9 implied the labyrinth developmental impairment, resulting in placental dysfunction.

In the labyrinth layer, the close juxtaposition of foetal and maternal blood circulations ensures efficient exchange between foetus and mother, and the mature BPB prevents the transmission of many toxicants from the maternal to the foetal circulation [46]. We found that exposure to Ti_3_C_2_ nanosheets could affect the structure of the placenta, lead to the reduction of the labyrinth area, and the placental efficiency. Defects in the labyrinth layer result in poor oxygen, nutrient diffusion, and defective BPB, which are associated with foetal growth restriction, intrauterine growth restriction (IUGR), or other pregnancy-related diseases [47]. Between the foetal blood vessel and maternal blood sinuses, syncytiotrophoblast was observed to be arising from the fusion of appropriate trophoblast cells. We quantified proliferating spongiotrophoblasts and endothelial cells in placentae at various doses exposure groups. Results showed that Ti_3_C_2_ nanosheet exposure increased trophoblast proliferation, combined with decreased endothelial cell migration in placentae, which may affect placental vascular maturation. Another major function of syncytiotrophoblast is to produce placental hormones to perform multiple functions of placenta and foetus development [48]. As a pregnancy-specific hormone produced by the syncytiotrophoblast, high or low hCG concentration is a marker of subsequent clinical manifestation of preeclampsia [49]. Abnormal levels of the PIGF angiogenic factor and the sFlt-1/PIGF ratio are often associated with several pregnancy disorders, such as placenta accrete and preeclampsia [50, 51]. This alteration of hormones is similar to the results observed in our study, indicating the dysregulated placental hormone secretion induced by Ti_3_C_2_ nanosheets. Unexpectedly, these impairments of the placental structure and hormone secretion did not affect the reproductive ability of maternal mice, but it caused abnormal neurobehavior and pathological changes in the cerebral hippocampus and cortex in offspring. As the nutritional support organ of foetus, the placental metabolism and metabolites transport efficiency determines foetal neurodevelopment critically. Thus, we further investigated the metabolome of the placenta.

As the final production of the genome, transcriptome, and proteome, metabolome can supply worthy mechanistic information related to the phenotype of the organism. Metabolomics has been an effective analytical tool for indicating the toxicological effects and exploring the mechanistic of nanomaterials [52, 53]. In our study, the metabolites profiles were verified in the placenta after Ti_3_C_2_ nanosheet exposure, demonstrating a significant accumulation of amino acids, saturated fatty acids, unsaturated fatty acids, and TCA cycle metabolites, etc. Among these metabolites, amino acids-the principal nutrient supplements, were especially important for normal foetal growth. The high concentration of placental amino acids, particularly glutamic acid and histidine enhances the nutrient transport to the foetus, can functionally adapt to satisfy foetal normal growth at late pregnancy period [54, 55]. As a result of this adaptation, the foetus stays within a normal birth weight range at term, which is in accordance with our results. Besides, the long-chain unsaturated fatty acids are essential for the proper neurological development of the foetus [56]. As a monounsaturated omega-9 fatty acid, a high concentration of maternal erucic acid may influence the risk of small gestational age infants [57]. With enriched sphingomyelin, nervonic acid was essential for the neural development of the new-born [58]. As a long-chain polyunsaturated fatty acids (LCPUFAs), DHA derived from linoleic and α-linolenic acids is also necessary for the development of the foetal nervous system [59]. Previously studies suggest learning ability may be impaired by the reduction in accumulation of sufficient DHA during the intrauterine period [60, 61]. The high accumulation of placental metabolites could be associated with transport issues, deficient metabolic enzymes, the activity of secondary pathways, or clearance impairment [22, 62]. In our lipid metabolic pathway analysis, biosynthesis of unsaturated fatty acids was significantly upregulated, while linoleic acid metabolism, glycerolipid metabolism, and sphingolipid metabolism were significantly downregulated. The placental lipid metabolism is a determinant of the quantity and composition of fatty acids delivered to the foetus [22]. Maternal conditions, such as toxins exposure have now been shown to influence this process and thus impair placental fatty acid transfer and foetal neurodevelopment. During embryonic development, the proliferation and specificity of ventricular zone cells are easily affected by many factors. The normal development ventricular zone cells play a function in determining the quantity and variety of foetal neocortical neurons [63]. As the centra of the nervous system, the cerebral neocortex is involved in a series of bioactivity in higher organisms, such as learning, memory, emotion, etc. A negative placental reaction to toxins might leave a permanent mark on the foetal central nervous system, leading to neurological like autism, schizophrenia, and motor disturbances in the offspring as they reach adulthood [64–66].

## Conclusions

In summary, our results suggested that Ti_3_C_2_ nanosheets accumulated in the uterus and placenta after maternal Ti_3_C_2_ nanosheets exposure. Those accumulations in the intrauterine environment barely affected the reproductive ability of female mice, while the neurotoxic effects in offspring were obvious. The results that decreased maternal body gain, organ coefficient of the uterus, and intrauterine bleeding demonstrated the intrauterine environment is impaired after maternal Ti_3_C_2_ nanosheets exposure. Further research revealed that the abnormal development and function of the placenta, as well as altered placental lipid metabolism, might be a major contributor to the neurodevelopmental impairment observed in offspring. These data provide an experimental basis for the harmful effects of Ti_3_C_2_ nanosheets exposure during early pregnancy, which can provide a new insight for investigating reproductive toxicology and neurotoxicity. Besides, our study suggested that evaluation of the effects of environmental factors on pregnancy should not only be limited to the observation of pregnancy outcome but also pay attention to the long-term effects in offspring, particularly neurodevelopment.

## Supplementary Information

**Additional file 1. Fig S1.** The development of placental labyrinth. **(a)** Histological section and immunohistochemical staining for BMP2 (scale bars = 200 μm and 20 μm) on GD8 in the vehicle control and 2.5 mg/kg group. EPC, ectoplacental cone; M, mesometrial side; AM, antimesometrial side. **(b)** Histological section and immunohistochemical staining for CK8 (scale bars = 200 μm and 20 μm) on GD9 in the vehicle control and 2.5 mg/kg groups. Ch, chorion

## Data Availability

All the original data are available upon reasonable request for correspondence authors.
