# Supplementary figures and images for: Exposure to two-dimensional ultrathin Ti3C2 (MXene) nanosheets during early pregnancy impairs neurodevelopment of offspring in mice
Source: J Nanobiotechnology. 2022 Mar 5;20:108. doi: 10.1186/s12951-022-01313-z (PMC8898431; doi:10.1186/s12951-022-01313-z)

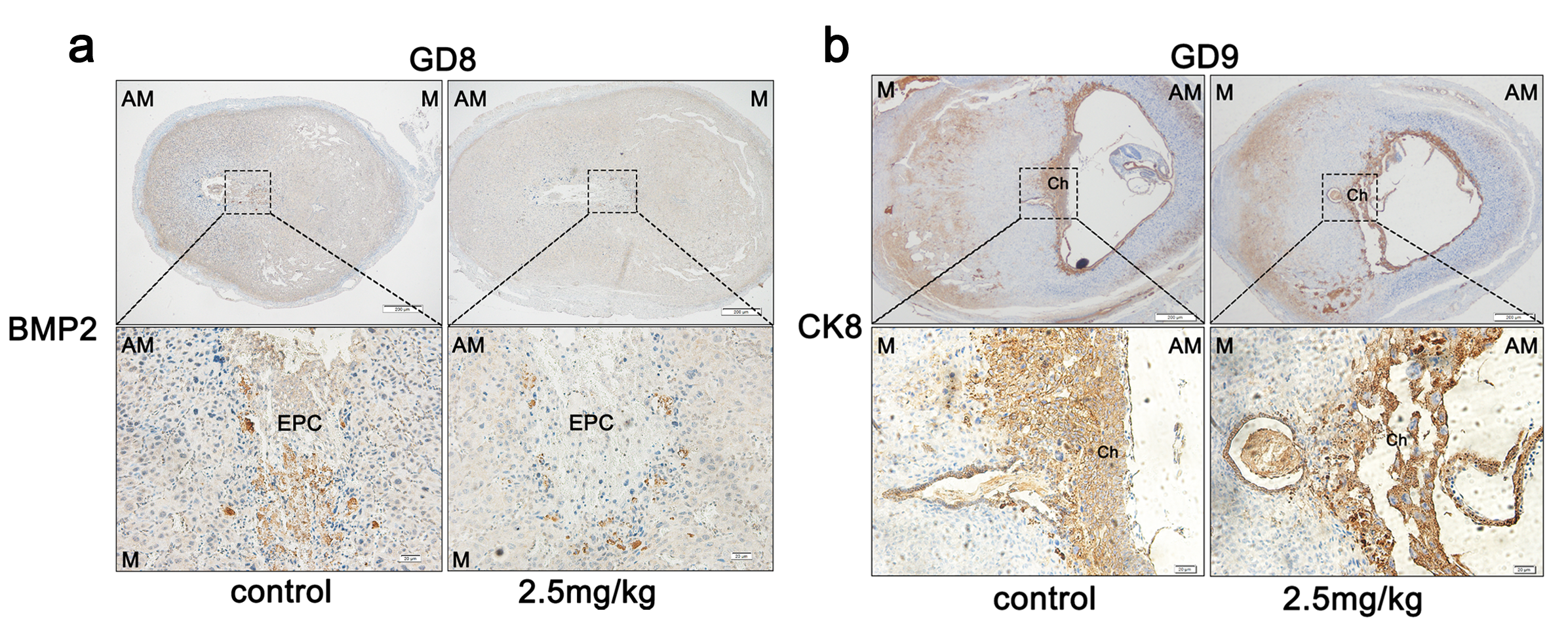

Supplement: Supplementary file 1 — Additional file 1. Fig S1. The development of placental labyrinth. (a) Histological section and immunohistochemical staining for BMP2 (scale bars = 200 μm and 20 μm) on GD8 in the vehicle control and 2.5 mg/kg group. EPC, ectoplacental cone; M, mesometrial side; AM, antimesometrial side. (b) Histological section and immunohistochemical staining for CK8 (scale bars = 200 μm and 20 μm) on GD9 in the vehicle control and 2.5 mg/kg groups. Ch, chorion [file 12951_2022_1313_MOESM1_ESM.tif]
